# Porcine extraintestinal pathogenic *Escherichia coli* delivers two serine protease autotransporters coordinately optimizing the bloodstream infection

**DOI:** 10.3389/fcimb.2023.1138801

**Published:** 2023-02-16

**Authors:** Xinming Pan, Rong Chen, Yating Zhang, Yinchu Zhu, Jin Zhao, Huochun Yao, Jiale Ma

**Affiliations:** ^1^ Ministry of Education (MOE) Joint International Research Laboratory of Animal Health and Food Safety, College of Veterinary Medicine, Nanjing Agricultural University, Nanjing, China; ^2^ Key Lab of Animal Bacteriology, Ministry of Agriculture, Nanjing, China; ^3^ Office International Des (OIE) Reference Lab for Swine Streptococcosis, Nanjing Agricultural University, Nanjing, China; ^4^ Institute of Animal Husbandry and Veterinary Sciences, Zhejiang Academy of Agricultural Sciences, Hangzhou, China; ^5^ Department of Animal Science, Yuxi Agriculture Vocation-Technical College, Yuxi, China

**Keywords:** extraintestinal pathogenic *E. coli*, bloodstream, serine protease autotransporter, immunomodulation, mucin-like glycoprotein

## Abstract

Extraintestinal pathogenic *Escherichia coli* (ExPEC) is one of the leading causes of bloodstream infections in a broad spectrum of birds and mammals, thus poses a great threat to public health, while its underlying mechanism causing sepsis is not fully understood. Here we reported a high virulent ExPEC strain PU-1, which has a robust ability to colonize within host bloodstream, while induced a low level of leukocytic activation. Two serine protease autotransporters of Enterobacteriaceae (SPATEs), Vat^PU-1^ and Tsh^PU-1^, were found to play critical roles for the urgent blood infection of strain PU-1. Although the Vat and Tsh homologues have been identified as virulence factors of ExPEC, their contributions to bloodstream infection are still unclear. In this study, Vat^PU-1^ and Tsh^PU-1^ were verified to interact with the hemoglobin (a well-known mucin-like glycoprotein in red blood cell), degrade the mucins of host respiratory tract, and cleave the CD43 (a major cell surface component sharing similar O-glycosylated modifications with other glycoprotein expressed on leukocytes), suggesting that these two SPATEs have the common activity to cleave a broad array of mucin-like O-glycoproteins. These cleavages significantly impaired the chemotaxis and transmigration of leukocytes, and then inhibited the activation of diverse immune responses coordinately, especially downregulated the leukocytic and inflammatory activation during bloodstream infection, thus might mediate the evasion of ExPEC from immune clearance of blood leukocytes. Taken together, these two SPATEs play critical roles to cause a heavy bacterial load within bloodstream *via* immunomodulation of leukocytes, which provides a more comprehensive understanding how ExPEC colonize within host bloodstream and cause severe sepsis.

## Introduction

Extraintestinal pathogenic *Escherichia coli* (ExPEC) is responsible for 80%–90% of community acquired urinary tract infections (Ejrnaes, 2011) as well as 30% of bacteremia (Diekema et al., 2002), thus is one of the major agents of human diseases. In addition, ExPEC infects a broad spectrum of birds and mammals, such as causing sepsis and sudden death in swine and avian species (Dho-Moulin and Fairbrother, 1999; Ding et al., 2012), thereby is an important zoonotic pathogen to threaten public health. To cause sepsis, most virulent ExPEC strains have the ability to survive and proliferate within host bloodstream (Kaper, 2005). Although numerous virulence factors have been identified in diverse ExPEC pathotypes, the underlying mechanisms of ExPEC breaking through the barriers and evading immune clearance during bloodstream infection are incompletely understood.

Numerous serine protease autotransporters of enterobacteria (SPATEs) from class-2 have been identified in pathogenic *E. coli* to degrade a variety of mucins, thus play critical roles during the bacterial infection (Harrington et al., 2009; Ruiz-Perez and Nataro, 2014; Gibold et al., 2016), while related mechanisms involving in bloodstream infection have never been clarified in a sepsis isolate. Mucin is the major component of mucus layer widely presenting on the surface of epithelial tissues in the respiratory, urinary and genital tracts (Harrington et al., 2009; Navarro-Garcia et al., 2010; Gibold et al., 2016). In adherent-invasive *E. coli* (AIEC) strain LF82, the class-2 SPATE “Vat” promotes crossing of the intestinal mucus layer to cause Crohn’s disease (Gibold et al., 2016). Besides contributing to intestinal colonization, SPATEs “Pic” and “Tsh” are expressed during urinary tract infection of uropathogenic *E. coli* (UPEC), and significantly associated with the acute pyelonephritis (Heimer et al., 2004). Class-2 SPATEs also display a lectin-like activity with affinity to degrade diverse O-glycosylated mucin-like substrates, including the leukocyte surface O-glycoproteins (Henderson et al., 1999; Leyton et al., 2003; Ruiz-Perez et al., 2011). The cleavages of this type O-glycoproteins usually damage their vital roles in numerous cellular functions in leukocytes, thus result in immunomodulation (Szabady et al., 2009; Ruiz-Perez et al., 2011). These observations suggested that the SPATEs may mediate the immune evasion of bacterial pathogens from leukocytes, while there were no further studies providing solid evidences to verify that these functions contribute to the bacterial survival during bloodstream infection.

In this study, two class-2 SPATE encoding genes (*vat^PU-1^* and *tsh ^PU-1^*) of porcine ExPEC strain PU-1 were identified to be significantly upregulated in host blood but not in serum *in vitro*. Following works identified that these two SPATEs significantly interact with hemoglobin of red blood cell (RBC) for adhesion and spreading within bloodstream, and impair polymorphonuclear leukocytes’ (PMNs) functions *via* cleaving the mucin-like O-glycoproteins for immune evasion, which help to better understand how ExPEC colonizing within host bloodstream and cause severe sepsis.

## Materials and methods

### Bacterial strains and genetic manipulations

Bacterial strains and plasmids used in this study were listed in **Table S1**. PU-1 is an O2:K1 ExPEC strain (isolated from the blood of a piglet) causing acute sepsis in mouse infection model (Ma et al., 2020; Ma et al., 2021). All strains were grown on Luria-Bertani (LB) broth medium at 37°C with 180 rpm, supplemented with corresponding antibiotics, or isopropyl–D-thiogalactopyranoside (IPTG) when necessary. DNA amplification, ligation and electroporation were performed as previously described (Ma et al., 2018) unless otherwise indicated. Deletion mutants were constructed using the λ red mutagenesis method (Datsenko and Wanner, 2000), and the details of primers, restriction enzymes and fragments’ deletion have been listed in **Table S2**. All restriction and DNA-modifying enzymes were purchased from Thermo Fisher Scientific (Waltham, MA, USA) and performed according to the supplier instruction.

### Ethical approval statement

Porcine blood and mucus of the respiratory tract were collected from the healthy pigs of a slaughterhouse to perform the following studies. Blood from healthy donators was obtained from Jiangsu province Blood Center, and related experiments were approved by the Medicine Human Subjects committee of Jiangsu Province. Five-week-old female specific pathogen free (SPF) BALB/c mice were purchased from Yangzhou University (Comparative Medicine Center). All animal experiments were performed in strict accordance with the animal welfare standards of the Animal Research Committee Guidelines of Jiangsu Province (License Number: SYXK (SU) 2017-0007), and approved by the Ethics Committee for Animal Experimentation of Nanjing Agricultural University.

### Mouse infection assay

Ten mice in each group were challenged by intraperitoneal injection with the indicated strain at the designed doses and monitored for symptoms until 7 days post-infection. The negative-control group was challenged with an equal volume of sterile PBS. To evaluate bacterial proliferation *in vivo*, the bacterial load assay was conducted. Five mice in each group were inoculated with 1 × 10^6^ CFU/mouse of the indicated strain, the infected blood was harvested at the designed time of post-infection, and then serially diluted in PBS and plated on LB agar to enumerate the CFU.

### RNA isolation and RT-qPCR analysis

Total RNA was extracted with the E.Z.N.A. bacteria RNA isolation kit (Omega), and residual genomic DNA was then removed by digestion with DNase I (TaKaRa). The PrimeScript RT reagent kit (TaKaRa) was used for cDNA synthesis. The RT-qPCR was performed using SYBR premix Ex Taq (TaKaRa) with the gene-specific primers. The relative amount of target gene mRNA was normalized to the transcript of housekeeping gene *tus* (Ma et al., 2018), and the relative fold change was calculated by the threshold cycle (2^-ΔΔCT^) method. The reported values represented the mean ± SD of three independent RNA extractions.

### ELISA with hemoglobin

The recombinant His_6_-Vat^PU-1^ and His_6_-Tsh^PU-1^ proteins were purified by Ni-NTA Spin Columns (QIAGEN) from BL21 (DE3) carrying the recombinant pET-21a plasmid after IPTG induction. Human hemoglobin (Sigma-Aldrich) prepared at a concentration of 50 μg/mL in PBS were coated onto separate wells of a 96-well plate. The purified His_6_-Vat^PU-1^ and His_6_-Tsh^PU-1^ were added to each well for 1 h at 37°C, and then washed three times and incubated with the anti-His antibody (Abcam, 1:2000) at 37°C for 2 h. After thrice wash, the processed membranes were stained with the HRP conjugated secondary antibodies (Thermo Fisher, 1:2000) at 37°C for 1 h, and detected using the 3,3’-diaminobenzidine. The reaction was stopped after 30 min by addition of 1 M H_2_SO_4_, and absorbance was measured at 450 nm. Three independent experiments were performed, with four wells for every reaction in each experiment, and the values obtained were averaged.

### Mucin gel penetration assay and cleavage assay

The mucin gel penetration assay were used as previously described (Henderson et al., 1999; Silva et al., 2003). A solution containing 10% mucin of porcine respiratory tract and 0.3% agarose in HBSS was loaded into a 1-mL injection syringe, creating a mucous column. A 0.1 mL prepared bacterial cells (10 × 10^9^ cells/mL) were layered onto the mucin. The columns were incubated for 3 h at 37°C in a vertical position. Afterwards, five fractions (each one contains 0.2 mL) were collected from the button by applying gentle pressure. Each fraction was serially diluted and plated to the LB agar media for CFU enumerating. Western blot assays were performed to analyses the cleavage activity of SPATEs to mucin. The mucins were extracted from the mucus of porcine respiratory tract as previously described (Gibold et al., 2016). Degradation reactions were separated by 12% SDS-PAGE, and then transferred to PVDF membranes (Bio-Rad) for subsequent blocking, washing, incubating with the specific and secondary antibodies (using as the instructions of manufacturer), and detecting using the 3,3’-diaminobenzidine.

### Flow cytometry

Before incubating with the conjugated mAb of APC-CD43 (Invitrogen), PMNs were incubated with the human IgG to block Fc receptors. The prepared cells were incubated with SPATEs for the indicated times, and then stained by incubation with the dye-conjugated antibodies specific to the extracellular domain of host glycoproteins. The positive staining with antiCD16 and CD16b mAbs (R&D Systems) were selected as the low forward-scatter and high side-scatter characteristics, respectively, and then used to gate the neutrophils. The samples were analyzed in an Accuri C6 fow cytometer (BD Accuri)/fluorescence-activated cell sorter (FACS), and analyzed using the CFlow plus software (BD Accuri).

### PMNs chemotaxis and transendothelial migration assays

Chemotaxis and transmigration assays were performed according to previously described protocols (Yamamoto et al., 2000; Ruiz-Perez et al., 2011). For the chemotaxis assay, 3 × 10^5^ calcein-stained human PMNs were incubated with the indicated protein in the upper chamber of Transwell, and 100 mM of IL-8 (MedChemExpress) or 100 nM of fMLP (N-formyl-methionineleucine-phenylalanine, MedChemExpress) was added to the lower chamber as chemoattractant. After 4 h incubation, the PMNs that had migrated toward the chemoattractant were collected for counting with a Fluoroskan fluorometer. For transendothelial migration assays, human brain microvascular endothelial cells (HBMECs) were seeded on the inserts of Transwells at a concentration of 2 × 10^4^ cells/well, and cultured until the monolayers were confluent (~ 4 days). Afterwards, similar operating steps were performed as the chemotaxis assays.

### Detection of blood indicators

The blood samples were analyzed by a Blood RT (Routine Test) machine in the Animal Hospital of Nanjing Agriculture University. The whole bloods of mice infected with the indicated bacterial strains were collected, and centrifuged to get the sera. The mouse interleukin 6 and 8 ELISA test kits (HUYU biological technology Co Ltd, shanghai) were used as the instructions of manufacturer to detect the levels of interleukin 6 and 8 release in the sera.

### Statistical analysis

Statistical analyses were performed using Prism 8.0 (GraphPad), and the full details were described below. Two-way ANOVA was used for the qRT-PCR assay, One-way ANOVA was performed for the results of bacterial survival assay, blood routine test, IL-6 and IL-8 release, PMNs chemotaxis and transmigration. For infection experiments, survival data were analyzed with the log rank test. For all tests, a *P* value < 0.05 were considered statistically significant, and all data were shown as mean ± SD.

## Results

### Low levels of leukocytic activation were induced by ExPEC strain PU-1 during the severe bloodstream infection

**Figure 1:**
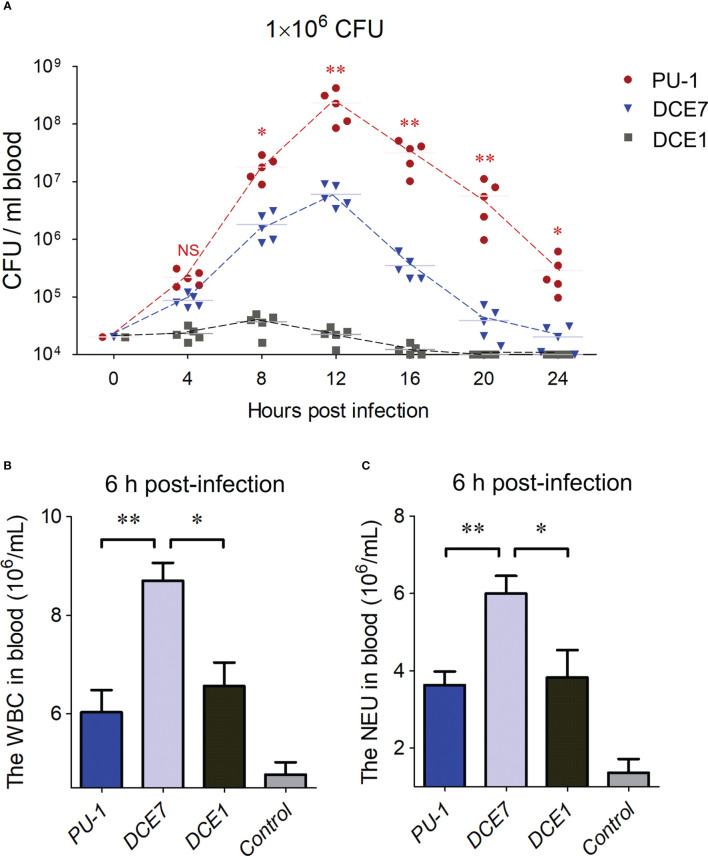
ExPEC strain PU-1 caused a heavy bacterial load in infected mouse blood. **(A)** The curves showed the bacterial loads in bloods from the mice challenged with different *E. coli* strains until 24 h post infection. The ExPEC strains DCE7 and DCE1 were used as controls here, respectively. Statistical significance of PU-1 infection group was determined by a one-way ANOVA test based on comparisons with the DCE7 infection group (**P < 0.01, *P < 0.05). **(B, C)** Blood routine testing detected the white blood cell (WBC) and neutrophil cell (NEU) counts of peripheral blood from the mice infected with indicated ExPEC strains at the 6 h post-infection. Statistical significance was determined by a one-way ANOVA test based on comparisons with the wild-type group (**P < 0.01, *P < 0.05). NS, no significance.

PU-1 is an O2:K1 ExPEC strain causing acute sepsis in mouse infection model (Ma et al., 2020; Ma et al., 2021). The curve of blood bacterial loads in mice infected by strain PU-1 showed the maximum value (more than 10^8^ CFU/mL) at 12 h post-infection (**Figure 1A**), which was remarkably higher than the infection groups of virulent ExPEC strain DCE7 from phylogenetic group D (Zhu et al., 2017), and strain DCE1 from group A. Furthermore, the blood of mice (with obvious clinical symptoms: tremble, orbital hemorrhage, anorexia, ataxia, anaesthesia et al.) in PU-1 infection group showed a significantly slighter decrease in bacterial loads after 12 h post-infection than the mice challenged with strains DCE7 and DCE1 (**Figure 1A**). The blood routine testing results showed that white blood cell (WBC) and neutrophil cell (NEU) counts of peripheral blood from the PU-1 infection group were significantly less than that of DCE7 infection group (**Figures 1B, C**) at the 6 h post-infection (no significant differences in blood bacterial loads between PU-1 and DCE7 infection groups at this point in time). These data suggested that strain PU-1 has the potential to modulate the host immune responses for its optimal blood infection *in vivo*.

### Two serine protease autotransporters, Vat^PU-1^ and Tsh^PU-1^, were identified to contribute to blood survival of strain PU-1 *in vitro*

**Figure 2:**
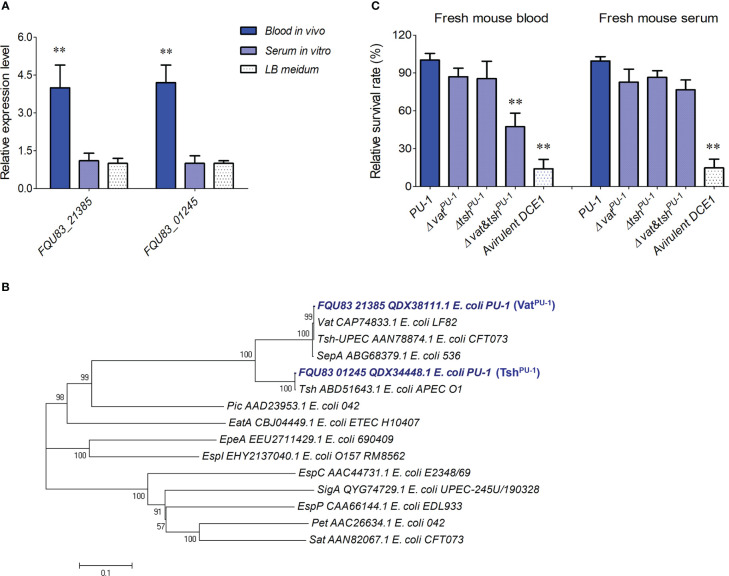
Identification of two serine protease autotransporters as the potential facilitators for optimal blood infection in strain PU-1. **(A)** The transcriptional changes of indicated genes in ExPEC strain PU-1 response to host blood and serum. The data were normalized to the housekeeping gene *tus* transcript. Mean values and SDs of triplicate samples are indicated. Statistical significance was determined by a two-way ANOVA test based on comparisons with the bacterial cells cultured in LB medium (**P < 0.01). **(B)** Phylogenetic analysis of SPATEs from *E coli*. A neighbor-joining tree (bootstrap n = 1000; Poisson correction) was constructed based on a ClustalW alignment of the amino acid sequences of SPATEs using the MEGA software version 5.0. **(C)** Incubations of indicated bacterial strains within fresh blood and serum. The survival rates were calculated by measuring the bacterial counts. The porcine ExPEC strain DCE1 from phylogenetic group A were used as a control here. Statistical significance was determined by a one-way ANOVA test based on comparisons with the wild-type group (**P < 0.01).

Comparative analysis based on our previous transcriptome data (Ma et al., 2020) found two special genes, *FQU83_21385* (chromosome encoding) and *FQU83_01245* (plasmid encoding), which encoded two serine protease autotransporters of enterobacteria (SPATEs), and showed the significant upregulation of more than 3 folds in animal blood but not in serum (**Figure 2A**). Phylogenetic analysis showed that the FQU83_21385 and FQU83_01245 belong to the class-2 of SPATEs (**Figure 2B**), and share highest sequence identities with the well-studied virulence factors Vat homologue of strain LF82 and Tsh homologue of strain APEC O1, respectively, thereby were redesignated as Vat^PU-1^ and Tsh^PU-1^ in strain PU-1. To explore whether these two SPATEs involving in ExPEC’s bloodstream survival, non-polar deletion mutant strains were constructed under the background of wild-type strain PU-1. The results of bacterial counting demonstrated that the inactivation of Vat^PU-1^ alone or Tsh^PU-1^ alone did not affect the bacterial survival in host blood (**Figure 2C**). It should be noted that mutant strain with double deletions of *vat^PU-1^* and *tsh^PU-1^* significantly decreased survival in host blood, while had no significant effect for the bacterial survival in host serum (**Figure 2C**). These results suggested that at least one SPATE in strain PU-1 was required to resist the clearance mediated by immunocytes within blood, while was not involved in resistance to the bactericidal effects of serum.

### Vat^PU-1^ and Tsh^PU-1^ contributed to bloodstream infection

**Figure 3:**
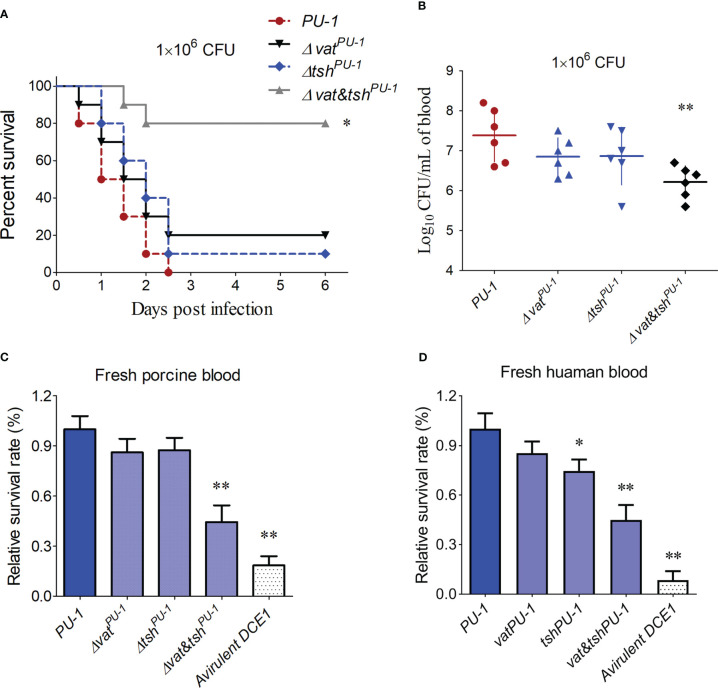
Mouse infection assay identified the pathogenic roles of VatPU-1 and TshPU-1. **(A)** Effect of *vat^PU-1^* or *tsh^PU-1^* deletion on strain PU-1 pathogenicity. Survival curve of mice infected with 1 × 10^6^ CFU/mouse bacteria (ten mice per group). **(B)** Systemic infection experiment was conducted to assess bacterial load in mouse blood. Bacterial reisolation from the blood at 16 h post-inoculation was quantified by plate count. **(C, D)** Porcine and human bloods were then used to assess the bacterial survival of indicated ExPEC strains. The survival rates were calculated by measuring the bacterial counts. Statistical significance was determined by a one-way ANOVA test (**P < 0.01, *P < 0.05).

An animal infection test using the BALB/c mice was employed to further certify the pathogenic roles of these two SPATEs during bloodstream infection. As shown in **Figure 3A**, the mice infected by the Δ*vat&tsh^PU-1^*, but not the single deletion mutants Δ*vat^PU-1^* and Δ*tsh^PU-1^*, showed a significantly higher survival rate (80%), compared with the 100% death of mice infected by the wild-type strain. Consistently, only the double deletions of *vat^PU-1^* and *tsh^PU-1^* significantly attenuated the bacterial loads in mice blood compared with the wild-type strain (**Figure 3B**). Porcine and human bloods were then used to assess the survival rates of indicated ExPEC strains. The results demonstrated that the mutant strain with double deletions of *vat^PU-1^* and *tsh^PU-1^* significantly decreased survival in both of these two types of blood (**Figures 3C, D**), while the inactivation of Vat^PU-1^ alone or Tsh^PU-1^ alone did not affect the bacterial survival here. These data indicated that Vat^PU-1^ and Tsh^PU-1^ are critical for the full virulence and blood infection in ExPEC strain PU-1.

### Vat^PU-1^ and Tsh^PU-1^ interacted and degraded mucin-like O-glycoproteins

**Figure 4:**
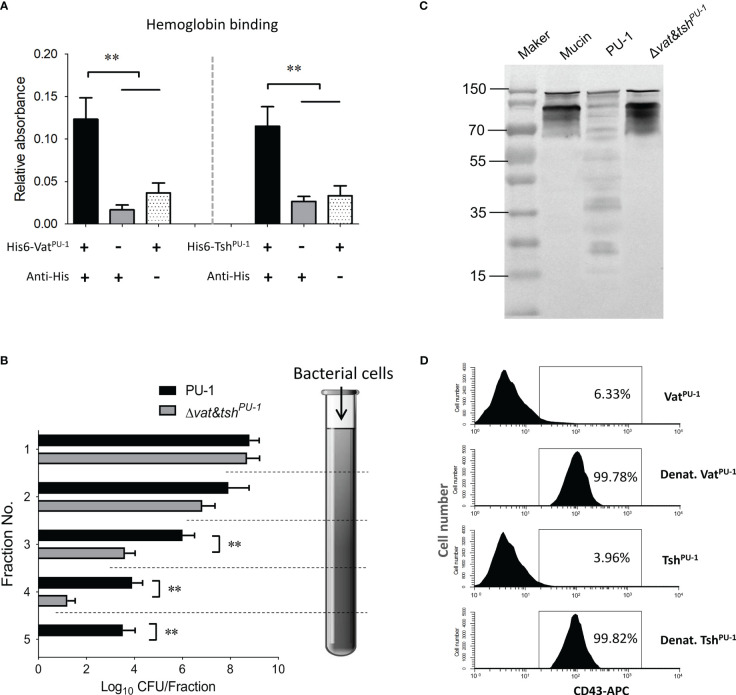
Vat^PU-1^ and Tsh^PU-1^ bound to and cleaved diverse O-glycosylated mucin-like proteins. **(A)** SPATEs bind to the hemoglobin. Human hemoglobin was coated onto a 96-well microplate and incubated with the purified proteins. Binding was detected by an indirect ELISA. **(B)** The assessment of mucin degradation by using a column penetration assay. Quantification of indicated ExPEC strains in fractions eluted from columns filled with gel-forming mucus (1 to 5 fractions: top to bottom of the column). **(C)** Mucin extracted from porcine respiratory tract was incubated overnight with filtered culture supernatants of indicated ExPEC strains. Mucin degradation was observed by Western blot using an anti-MUC2 antibody. **(D)** The degradation mediated by Vat^PU-1^ and Tsh^PU-1^ to the extracellular domain of O-glycosylated mucin-like protein CD43 on human leukocytes. The PMNs were isolated from human blood and incubated with the purified Vat^PU-1^, Tsh^PU-1^, or denatured proteins at 37°C for 30 min. Flow cytometry was employed to analyze these samples using monoclonal antibodies against the extracellular domains of CD43. Flow cytometry data are representative of at least three independent experiments. **P value < 0.05, which is considered statistically significant.

Numerous class-2 SPATEs of pathogenic *E. coli* have been reported to bind to and cleave mucin-like proteins for optimal colonization (Henderson et al., 1999; Dutta et al., 2002; Leyton et al., 2003; Harrington et al., 2009), including Pic, Tsh, Vat, et al. In this study, both of Vat^PU-1^ and Tsh^PU-1^ were identified to have the abilities to bind to hemoglobin (**Figure 4A**), a well-known mucin-like glycoprotein widely presenting in red blood cells (RBCs). We then investigated the ability of Vat^PU-1^ and Tsh^PU-1^ to degrade mucus by using a column penetration assay. Unlike Δ*vat&tsh^PU-1^*, wild-type strain PU-1 penetrated through the entire mucus column (fractions 1 to 5, **Figure 4B**). In the fractions 3 and 4 (middle of the column), the efficiency of Δ*vat&tsh^PU-1^* was about two orders of magnitude lower than that of PU-1 strain at penetrating the mucus column. In order to investigate the mucinolytic activity of Vat^PU-1^ and Tsh^PU-1^, we examined the ability of related strains to hydrolyze porcine respiratory mucins by Western blotting. The cultural supernatant of wild-type strain PU-1 was able to degrade respiratory mucins unlike those of Δ*vat&tsh^PU-1^* (**Figure 4C**). Overall, these results suggest that Vat^PU-1^ and Tsh^PU-1^ promote the bacterial penetration through the mucus layer by altering the gel-forming mucins.

SPATEs have been confirmed to target a broad range of mucin-like glycoproteins present not only on the RBCs and epithelial cells, but also on the surface of leukocytes. To determine whether Vat^PU-1^ and Tsh^PU-1^ cleaved such proteins, the CD43, a major cell surface glycoprotein expressed on leukocytes, was addressed. We performed flow cytometry analyses of human PMNs treated with purified Vat^PU-1^, Tsh^PU-1^ and denatured proteins to further confirm the CD43 cleavage on cell surface. Staining with anti-CD43 mAbs revealed that the extracellular CD43 was present on the surfaces of PMNs after treatment with denatured Vat^PU-1^ and denatured Tsh^PU-1^ (**Figure 4D**), while was cleaved, becoming undetectable on cells treated with purified Vat^PU-1^ and Tsh^PU-1^. These results, coupled with the previous study verifying the cleavages of CD44, CD45, CD93 and CD162 by the Tsh homologue from Avian Pathogenic *E. coli* (APEC) (Ruiz-Perez et al., 2011), suggested that Vat^PU-1^ and Tsh^PU-1^ have the potential to cleave diverse O-glycosylated mucin-like proteins located on peripheral blood leukocytes, which may facilitate the bacterial survival within host bloodstream.

### Vat^PU-1^ and Tsh^PU-1^ impaired the PMNs’ chemotaxis and transmigration

**Figure 5:**
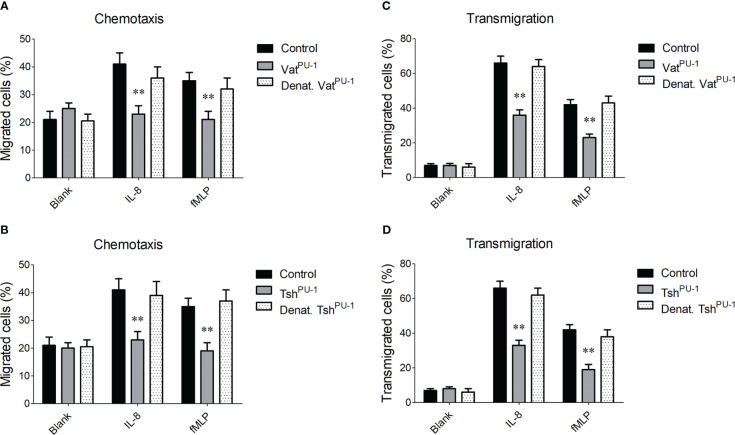
The impairment mediated by Vat^PU-1^ and Tsh^PU-1^ to the PMNs’ chemotaxis and transmigration through endothelial cell monolayers. **(A, B)** Assessment of PMNs’ chemotaxis. The calcein- labeled PMNs were treated with indicated proteins, or PBS vehicle control in the upper chamber of Transwell; IL-8 or fMLP were added to the lower chamber. Penetration of cells through the membrane was measured after 4 h fluorometrically. **(C, D)** Assessment of PMNs’ transmigration. The preincubated PMNs were applied to the upper chamber of Transwell supporting HBMEC, and transmigrated PMNs were enumerated as mentioned before. Statistical significance was determined by a one-way ANOVA test based on comparisons with the control group (** *P*<0.01). Error bars represent the SDs for three independent experiments.

We next explored whether the cleavage of mucin-like glycoproteins mediated by Vat^PU-1^ and Tsh^PU-1^ disturb the function of peripheral blood leukocytes during ExPEC infection. Here chemotaxis and transmigration assays of PMNs were performed according to previously described protocols (Ruiz-Perez et al., 2011; Ma et al., 2018). For the chemotaxis assays, IL-8 or fMLP within the lower chamber of Transwell were used to stimulate the translocation of PMNs through an abiological membrane from the upper chamber, and PMNs that migrated in the lower chamber were measured. The movements of PMNs stimulated by IL-8 and fMLP were significantly enhanced compared with the untreated PMNs in this model (**Figure 5A**), while incubation with the Vat^PU-1^ but not the denatured Vat^PU-1^ was significantly reduced the activated movement to the similar levels with the untreated PMNs. Similar results were also observed when the PMNs were incubated with the Tsh^PU-1^ (**Figure 5B**). These observations suggested that both two SPATEs can impair the PMNs’ chemotaxis. For the transmigration assays, the PMNs within the upper chamber of Transwell were stimulated to transmigrate through the endothelial cell monolayers, and PMNs in the lower chamber were measured after 4 h incubation. Expectedly, the transmigration of PMNs incubated with Vat^PU-1^ or Tsh^PU-1^ was significantly reduced compared with the buffer alone as negative control (**Figures 5C, D**); denaturation of Vat^PU-1^ or Tsh^PU-1^ by heating completely abolished the above effects. Taken together, these observations suggest that the PMNs’ functions were significantly impaired by the interaction with Vat^PU-1^ and Tsh^PU-1^ during the bloodstream infection of ExPEC.

### Vat^PU-1^ and Tsh^PU-1^ modulated the host immune responses *in vivo*

**Figure 6:**
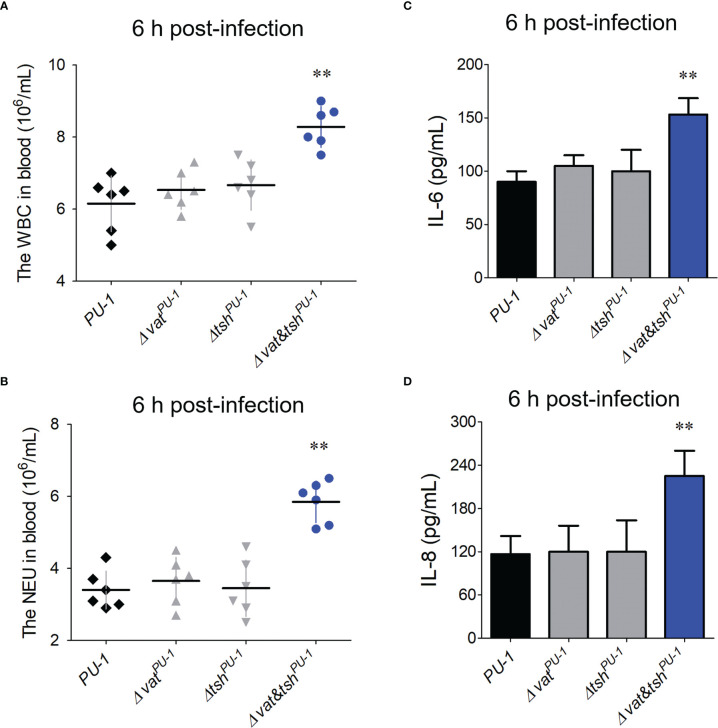
The assessment Vat^PU-1^ and Tsh^PU-1^ in modulation of host immune responses during blood infection. **(A, B)** Blood routine testing detected the white blood cell (WBC) and neutrophil cell (NEU) counts of peripheral blood from the mice infected with indicated ExPEC strains at the 6 h post-infection. **(C, D)** Detection of IL-6 and IL-8 levels in bloods from the mice infected with indicated ExPEC strains at the 6 h post-infection. Statistical significance was determined by a one-way ANOVA test based on comparisons with the wild-type group (**P < 0.01).

Further study managed to investigate the potential modulations of SPATEs to the immune responses in a mouse infection model. Blood routine testing results showed that white blood cell (WBC) and neutrophil cell (NEU) counts of peripheral blood from Δ*vat&tsh^PU-1^* infection group were significantly higher than that of wild-type infection group (**Figures 6A, B**). The cytokines’ detection was then performed. Compared with wild-type strain, the inactivation of Vat^PU-1^ alone or Tsh^PU-1^ alone did not affect the release levels of IL-6 and IL-8 in blood (**Figures 6C, D**). However, the double deletion mutant Δ*vat&tsh^PU-1^* induced the significantly higher levels of IL-6 and IL-8 at the 6 h post-infection (no significant differences in blood bacterial loads compared with the wild-type at this point in time), indicating that a greater inflammatory response was activated compared with the wild-type infection. These data suggested that Vat^PU-1^ and Tsh^PU-1^ coordinately attenuate the host immune responses, thus may mediate the evasion of ExPEC strain PU-1 from the immune clearance of blood leukocytes.

## Discussion

Although numerous virulence factors have been identified in diverse ExPEC pathotypes, the underlying mechanisms of ExPEC causing bloodstream infection is incompletely understood. ExPEC strain PU-1 showed a robust ability in survival within the blood, and this peculiarity may contribute to better show the full picture of ExPEC blood infection, suggesting that this strain deserves to be an excellent platform for further study.

In strain PU-1, the encoding genes of Vat^PU-1^ and Tsh^PU-1^ were significantly upregulated in host blood but not in serum *in vitro*, suggesting that the contact with host blood cells may be one of the necessary conditions for their transcriptional activation. Furthermore, inactivation of Vat^PU-1^ alone or Tsh^PU-1^ alone did not affect the bacterial survival in host blood, suggesting that these two SPATEs of strain PU-1 have obvious compensatory effects on each other, while they do not seem functionally redundant, as the encoding features of genes located in chromosome and plasmid may regulated by different mechanisms. Otherwise, the above analyses partially explain why we could not screen these two SPATEs as blood colonization factors in strain PU-1 through high-throughput technologies in previous studies (Ma et al., 2020; Ma et al., 2021). Although these studies have identified the carbon central metabolism, anaerobic respiratory chains, *de novo* biosynthetic pathways of nucleotide, extracellular polysaccharide biosynthesis and iron uptake playing critical roles for heavy bacterial load within bloodstream, the underlying mechanisms of strain PU-1 escaping from the host immune clearance *via* the interaction with the blood components (including RBCs, leukocytes, lymphocytes, et al.) were still incompletely understood. In this study, the roles of Vat^PU-1^ and Tsh^PU-1^ interacting with hemoglobin, mucus and leukocytes during blood infection were revealed, which filled in the potential loopholes of our previous screening strategies.

When proliferating in host blood, bacteria not only have to resist the bactericidal effects of serum, but also evade from the clearance of immune cells (including diverse leukocytes and lymphocytes) (Huja et al., 2014; Ma et al., 2018). The mucin-like glycoproteins are widely present on the surface of leukocytes, and play critical roles for the immune modulation (Ruiz-Perez et al., 2011; Ruiz-Perez and Nataro, 2014). Several homologues of class-2 SPATE have been reported to efficiently cleave leukocyte surface glycoproteins involved in diverse immune functions (Ruiz-Perez et al., 2011; Ruiz-Perez and Nataro, 2014), such as the cleavages of Pic and Tsh homologues to CD43, CD44, CD45, CD93, CD162, PSGL-1, and fractalkine, while the contributions of these phenotypes to the bacterial blood infection *in vivo* have never been verified. Among the most abundant mucin-like leukocyte surface glycoproteins, CD43 is widely expressed on nearly all lineages of hematopoietic cells (Rosenstein et al., 1999). The adhesion of leukocytes to neighboring cells can be prevented by CD43, which facilitate the leukocytes’ migration in response to chemokine attraction, while extra processing in CD43 mediated by pathogens may disrupt this native function (Manjunath et al., 1993; Seveau et al., 2000). Indeed, several secreted mucinases from diverse pathotypes of *E. coli* have been confirmed to inhibit PMNs’ chemoattraction and function by cleavage of CD43 (Szabady et al., 2009; Ruiz-Perez et al., 2011). Our study identified the cleavages of CD43 by the homologues of Vat and Tsh from strain PU-1, which is consistent with the previous findings of Tsh homologue in APEC strain (Ruiz-Perez et al., 2011). Cleavage of these diverse substrates from leukocyte surface could result in paralysis of the leukocyte-mediated response for optimal bloodstream infection.

The interactions with pathogens are not only mediated by immune cells during blood infection, but also widely occur in RBCs and platelets. Mucin-like glycoproteins widely present on the surface of nearly all lineages of hematopoietic cells (Ruiz-Perez et al., 2011; Ruiz-Perez and Nataro, 2014), including RBCs. By virtue of their ability to adhere glycoproteins, both the secreted and surface located “Tsh/hbp” SPATEs were found to agglutinate RBCs and bind to hemoglobin and extracellular matrix proteins (such as collagen IV and fibronectin), and these interactions were independent of its protease activity (Provence and Curtiss, 1994; Stathopoulos et al., 1999; Kostakioti and Stathopoulos, 2004). Our data verified that both of Vat^PU-1^ and Tsh^PU-1^ have the ability to bind to hemoglobin (**Figure 4A**), further works need to study the contributions of this phenotype to the bacterial blood infection. Several SPATEs have been reported to degrade the bound hemoglobin, and subsequently bind the released heme, thus contribute as an iron source generator (Otto et al., 1998; Otto et al., 2002). Numerous studies have confirmed the critical roles of iron uptake during bacterial infection within host bloodstream (Russo et al., 2001; Torres et al., 2001).

In summary, high virulent ExPEC strain PU-1 delivers two SPATEs to interact with the RBCs and blood leukocytes, which play critical roles to cause a heavy bacterial load within bloodstream by impairing PMNs’ functions *via* mucin-like O-glycoproteins’ cleavages. These findings provide a more comprehensive understanding how ExPEC colonizes within host bloodstream and causes severe sepsis.

## Data availability statement

The original contributions presented in the study are included in the article/**Supplementary Materials**. Further inquiries can be directed to the corresponding author.

## Ethics statement

The animal study was reviewed and approved by Animal Research Committee Guidelines of Jiangsu Province (License Number: SYXK (SU) 2017-0007).

## Author contributions

XP, JM, and HY: conceived and designed the experiments. XP and RC: performed the experiments. XP, YatZ, YinZ and JM: analyzed the data. JM and HY: reviewed the study. XP and JM: wrote the manuscript. All authors contributed to the article and approved the submitted version.
