# Supplementary material for: Porcine extraintestinal pathogenic Escherichia coli delivers two serine protease autotransporters coordinately optimizing the bloodstream infection
Source: Front Cell Infect Microbiol. 2023 Feb 16;13:1138801. doi: 10.3389/fcimb.2023.1138801 (PMC9978103; doi:10.3389/fcimb.2023.1138801)
Supplement: Supplementary file 1 [file Table_1.docx]

**Table S1** Summary of bacterial strains and plasmids for this study.

| Strain or plasmid | Characteristic(s) | | Source |
| --- | --- | --- | --- |
| **Strains** | | | |
| PU-1 | | Virulent ST95 O2:K1 ExPEC strain causing severe sepsis in mammal | Clinical isolation |
| DCE7 | | Virulent ExPEC strain isolated from the cerebrospinal fluid of a piglet with meningitis | Laboratory storage |
| DCE1 | | ExPEC strain isolate from the lung of a diseased piglet | Laboratory storage |
| Δ*vat^PU-1^* | | Deletion mutant of *vat* with PU-1 background | This study |
| Δ*tsh^PU-1^* | | Deletion mutant of *tsh* with PU-1 background | This study |
| Δ*vat&tsh^PU-1^* | | Deletion mutant of *vat* and *tsh* with PU-1 background | This study |
| *E. coli* DH5α | | Cloning host for maintaining the recombinant plasmids | Invitrogen |
| *E. coli* BL21 plysS | | Host for expressing the recombinant proteins | Invitrogen |
| **Plasmids** | |  |  |
| pET21a | | His-tag expressing vector, Amp^r^ | Laboratory stock |
| pET21a-Vat^PU-1^ | | pET21a(+) expressing Vat^PU-1^ protein | This study |
| pET21a-Tsh^PU-1^ | | pET21a(+) expressing Tsh^PU-1^ protein | This study |
| pKD46 | | Red recombinase expression plasmid | Laboratory stock |
| PKD4 | | pANTS derivative containing FRT-flanked kanamycin resistance | Laboratory stock |
| pCP20 | | TS replication and thermal induction of FLP synthesis | Laboratory stock |
