# Supplementary material for: Porcine extraintestinal pathogenic Escherichia coli delivers two serine protease autotransporters coordinately optimizing the bloodstream infection
Source: Front Cell Infect Microbiol. 2023 Feb 16;13:1138801. doi: 10.3389/fcimb.2023.1138801 (PMC9978103; doi:10.3389/fcimb.2023.1138801)
Supplement: Supplementary file 2 [file Table_2.docx]

**Table S2** Primers used for PCR amplification.

| Primer | Sequence(5'-3') |
| --- | --- |
| **General PCR for cloning** | |
| vat^PU-1^-F | GGCGGATCCGTCTCTGAACTAGCCCGAAGG |
| vat^PU-1^-R | GTGTCTAGAAAATGTTTGGCCCTGCAGTCT |
| tsh^PU-1^-F | GGCGGATCCGCAGGAAGTCTTGCGGGAACG |
| tsh^PU-1^-R | GTGTCTAGAATGTTCTCCCGCTGCATCCTT |
| **For Deletion** | |
| Del-vat^PU-1^-F | TCTATGGTTATTAATGTAACTTTGGAATATACGTTCCGGAATCATTTACTgtgtaggctggagctgcttc |
| Del-vat^PU-1^-R | TAGCGTTTATCGCATCATCTGTGTTGTATTTACCGAATGCAGAGCGTTCAatgggaattagccatggtcc |
| Del-tsh^PU-1^-F | CCGTTATGCCTGAGTAGTACTGATTTTATTTTTCTCAGGAGTAATTAAAAgtgtaggctggagctgcttc |
| Del-tsh^PU-1^-R | TTAGCGTTTATCGCATCATCTGTGTTGTATTTACCAAATGCAGAGCGTTCatgggaattagccatggtcc |
| **Primers for reverse transcription qPCR** | |
| tus-qPCR-F | TCGTAGACCGACTCAACACTA |
| tus-qPCR-R | TTACCTCCGGCAAAGAGAAC |
| vat^PU-1^-qPCR-F | GGAACTGGTCGGTGTTTACT |
| vat^PU-1^-qPCR-R | AAGACGGGAGCGTCATTATC |
| tsh^PU-1^-qPCR-F | CAGTAACGGACAGCTGACAAA |
| tsh^PU-1^-qPCR-R | CAGACCTGAACTGGTGCTAATC |
| **For checking** | |
| vat^PU-1^-Del-F | TGTAACTTTGGAATATACGTT |
| vat^PU-1^-Del-R | AGAATGAATAACGAATATTAG |
| tsh^PU-1^-Del-F | TTATGCCTGAGTAGTACTGAT |
| tsh^PU-1^-Del-R | TTAGCGTTTATCGCATCATCT |
| K1 | CCAGTCATAGCCGAATAGCCTC |
| K2 | CGGTGCCCTGAATGAACTGC |

Underlined showed restriction cutting sites; .
